# *Polygonum viviparum* L. induces vasorelaxation in the rat thoracic aorta via activation of nitric oxide synthase in endothelial cells

**DOI:** 10.1186/1472-6882-14-150

**Published:** 2014-05-07

**Authors:** Ming-Long Chang, Jung-Su Chang, Wen-Yu Yu, Khoot-Peng Cheah, Joe-Sharg Li, Hui-Wen Cheng, Chien-Ming Hu

**Affiliations:** 1 Emergency Department, Taipei Medical University Hospital, 252 Wu-Xing Street, Taipei 110, Taiwan; 2 School of Nutrition and Health Sciences, Taipei Medical University, Taipei Taiwan; 3 School of Pharmacy, College of Pharmacy, Taipei Medical University, Taipei Taiwan; 4 Department of General Medicine, School of Medicine, College of Medicine, Taipei Taiwan; 5 Medical University, 250 Wu-Xing Street, Taipei 110, Taiwan

**Keywords:** *Polygonum viviparum* L, Aorta, Vasorelaxation, cGMP, eNOS, HUVECs

## Abstract

**Background:**

In the past several decades, *Polygonum viviparum* L. (PV) was reported to have antibacterial, antiulcer, antioxidant, antitumor, anti-inflammatory, and antiarthritic properties. The anti-inflammatory pathway was recently elucidated through cytosolic nuclear factor E2-related factor 2 (Nrf2) activation and heme oxygenase (HO)-1 protein expression. PV is a perennial herb and widely distributed in high-elevation mountain regions, such as the Tibetan Plateau. In Tibetan traditional medicine, PV is usually used to boost the blood circulation to dissipate blood stasis. Therefore, this study focused on how PV improves the vascular circulation and acts on vascular tissues.

**Methods:**

In this study, we isolated aortas from Sprague-Dawley rats (male, weight about 250 ~ 350 g), and detected the effects of PV on phenylephrine (PE)-induced contraction and cyclic guanosine 3′,5′-monophosphate (cGMP) formation using aortic rings. In addition, human umbilical vein endothelial cells (HUVECs) were used to exam nitric oxygen (NO) synthase (NOS) activity by directly measuring NO production in the culture medium. Endothelial (e) NOS phosphorylation, and cytosolic Nrf2 and HO-1 expressions were measured using a Western blot analysis.

**Results:**

PV dose-dependently relaxed PE-induced contractions in endothelial-intact but not -denuded aorta. The concentration to produce 50% relaxation was 22.04 ± 1.77 μg/ml. PV-induced vasorelaxation was markedly blocked by pretreatment with *N*^G^-nitro-_L_-arginine methyl ester (L-NAME), an NOS inhibitor, methylene blue (MB), a guanylyl cyclase inhibitor, and hemoglobin, an NO scavenger. PV increased cGMP formation; however, this effect was also suppressed by co-pretreatment with l-NAME, MB, hemoglobin, and Ca^2+^-free medium. In HUVECs, PV increased NO formation, which was greatly attenuated by NOS inhibitors (L-NAME and L-NMMA) and by removing extracellular Ca^2+^ and chelating intracellular Ca^2+^ with BAPTA-AM. In addition, PV promoted eNOS phosphorylation, Nrf2 degradation, and HO-1 protein expression according to a Western blot analysis.

**Conclusions:**

The results suggest that PV possesses vasorelaxing action in an endothelium-dependent manner and works through activating Ca^2+^/calmodulin- dependent NO synthesis; when NO is released and then transferred to smooth muscle cells, NO activates guanylyl cyclase and increases cGMP formation, ultimately resulting in vasorelaxation. Thus, PV can be considered for application as a potential therapeutic approach for vascular-associated disorders.

## Background

*Polygonum viviparum* L. (PV), a perennial herb, belongs to the Polygonaceae family and is extensively distributed in high-elevation mountain region, including the Alps, Carpathians, Pyrenees, Caucasus Mountains, and the Tibetan Plateau [1,2]. The common names of PV are bistort, serpent-grass, and viviparous knotweed [3]. PV is a perennial herb that arises from a short, thickened rhizome that appears massive, distorted or uncinated. The stem, which ranges from 10 to 30 cm in height and terminates in a narrow, dense flowering spike, is simple, erect and smooth and bears few leaves. The best survey period lasts from approximately late June to early September [4]. In traditional folk medicine, PV is used to treat pharyngitis, dysentery, and gastrointestinal disorders [5]. The rhizome and root of PV are reported to possess excellent potency for curing bronchitis, piles, wounds, ulcers, vomiting, and biliousness [6-8]. From the literature over several decades, the major constituents of PV perhaps involve volatile oils [9], flavonoids, flavone glycosides [10-13], gallic acid, saponins, and tannins [14-16]. In addition, PV was demonstrated to possess efficacious bioactive effects, including antibacterial [11,17], antiulcer [7], antioxidant [8,18], antitumor [9,19], anti-inflammatory, and antiarthritic properties [20].

In our previous study, we demonstrated that PV has anti-inflammatory actions in macrophages, possibly acting through cytosolic nuclear factor E2-related factor 2 (Nrf2) activation to express heme oxygenase (HO)-1 protein [4]. On the other hand, especially in Tibetan traditional medicine, PV is usually used to boost the blood circulation to dissipate blood stasis [21]. Consequently, we wanted to determine how PV improves the vascular circulation, and what effect PV has on vascular tissues. In this study, the effect of PV on the thoracic aorta isolated from rats was examined.

## Methods

### Plant material

*Polygonum viviparum* L. (PV) was obtained from Tibet. Its authenticity was confirmed by Dr Shin-Ming Ku (Herbarium, Biodiversity Research Center, Academia Sinica, Taipei, Taiwan). The herb (PV 100 g) was extracted with 3 L of 2-propanol for 7 days, then the extract was filtered and centrifuged at 13 000 × *g* for 10 min. The extract supernatant was passed through a 0.22 μm sterile filter (Millipore, Billerica, MA, USA) and first concentrated using a vacuum rotary evaporator (Yamato, Tokyo, Japan) at 40°C. Normally, 8.76 g of dried powder could be obtained from 100 g of PV. The dried extract yield from the crude material was thus approximately 8.76% [4].

### Drugs and chemicals

Phenylephrine (PE), acetylcholine (Ach), *N*^G^-nitro-l-arginine methyl ester (l-NAME), *N*^G^-monomethyl-l-arginine acetate (l-NMMA), methylene blue (MB), hemoglobin (Hb), and all chemicals of the Krebs solution were from Sigma Chemical (St. Louis, MO, USA). Culture materials (M199 medium, fetal bovine serum (FBS), and trypsin-EDTA) were obtained from Life Technologies (Gibco, Grand Island, NY, USA). Endothelial cell growth supplement (ECGS) was purchased from Millipore (Billerica, MA, USA). A cyclic guanosine 3′,5′-monophosphate (cGMP) enzyme immunoassay kit was purchased from R & D System (Minneapolis, MN, USA). All other agents of cell culture were obtained from Sigma Chemical.

### Preparation of rat aorta and tension recording ex vivo

Male Sprague-Dawley rats weighing about 250 ~ 350 g were purchased from BioLASCO (Taipei, Taiwan). All animal procedures were approved by the institutional animal care and using committee of Taipei Medical University. Animals were housed in polycarbonate cages in a room at 22 ± 2°C on a 12-h light-dark cycle. The procedure described by Hu et al. [22] was used to isolate rat aortic rings. When the experiment began, the rats were sacrificed by exsanguination from the carotid artery under lose consicousness by knocking medulla; the thoracic aorta of rats was carefully removed, and the fat and tissue were dissected away in normal Krebs’ buffer at an adjusted pH of 7.4. The composition of this buffer was as follows: 118.5 mM NaCl, 4.8 mM KCl, 1.2 mM MgSO_4_, 1.2 mM KH_2_PO_4_, 2.5 mM NaHCO_3_, 11.1 mM glucose, and 2.5 mM CaCl_2_. The aorta was cut into rings about 5 mm long in Krebs buffer which was constantly gassed with 95% O_2_ + 5% CO_2_ at 37 ± 0.5°C. Two “L”-type stainless steel hooks were inserted into the aortic lumen; one side was fixed in the bottom bath and the other side was connected to a force transducer using a cotton thread. The aortic rings were equilibrated in Krebs buffer and maintained under a 1-g tension for 60 ~ 90 min, with three changes of buffer, before the experimental procedures began. Contractions were recorded isometrically via an iWorx FT-302 force transducer connected to an iWorx 304 T recorder (iWorx System, Dover NH, USA). In denuded aorta, the endothelium was removed by rubbing with a cotton ball, and the absence of ACh-induced relaxation was taken as an indicator of successful denudation. After PE-induced contraction and ACh-induced relaxation twice, PV (1, 3, 10, 30, 100 and 300 μg/ml) was treated after PE-induced contraction. The effects of PV as percentage of relaxation considering the maximum contraction elicited by phenylephrine in preparations with at least 80% of relaxation to ACh.

### Cyclic guanosine 3′,5′-monophosphate (cGMP) measurement

Rat aorta cGMP was analyzed using the method of Kauffman et al. [23]. Briefly, the aorta was immediately isolated from a rat, and cut into segments of about 20 mg/tissue. First, the rat aortic rings were pre-incubated in Krebs’ solution with 3-isobutyl-1-methylxathine (IBMX, 10 μM) for 5 min. Then, the aortic segments were placed in Ca^2+^-free Krebs’ (EGTA 2.5 mM) buffer or pretreatment inhibitors of l-NAME (50 μM) and MB (10 μM) for 10 min; then PV (100 μg/ml), sodium nitroprusside (SNP, 10 μM), and ACh (10 μM), as positive reagents, were added for a further 2 min. After incubation with PV and ACh, the aortic segments were rapidly frozen in liquid nitrogen and stored at -80°C until homogenized in 0.5 ml of 10% trichloroacetic acid using a motor-driven glass homogenizer. The homogenate was centrifuged at 10,000 × *g* for 5 min, and the supernatant was removed and extracted three times with 1.5 ml of water-saturated diethyl ether. The cGMP content was then assayed using enzyme immunoassay kits (R & D System, Minneapolis, MN, USA). Protein was measured by dissolving the trichloroacetic acid precipitate in 1 ~ 2 ml of 5 N NaOH followed by analysis using the method of Lowry et al. [24].

### Cell culture

Human umbilical vein endothelial cells (HUVECs, confluent second passage, P = 2) were purchased from the Bioresource Collection and Research Center (BCRC), Food Industry Research and Development Institute (Hsinchu, Taiwan). Cells were grown at 37 ± 0.5°C in a humidified 5% CO_2_ atmosphere in M199 medium (pH 7.4) supplemented with 10% FBS, 25 U/ml heparin, 30 μg/ml ECGS, 2 mM glutamine, 1.5 g/l NaHCO_3_, 10,000 units/l of penicillin, and 100 mg/l of streptomycin. Culture plates were coated with 1% gelatin before use. Confluent cells were detached by trypsin-EDTA (0.05%: 0.02%, v/v), and cells from passages 3 to 7 were used in the experiment.

### Determination of nitric oxide (NO) production

HUVECs (5 × 10^5^ cells/well) in 6-well plates were incubated with or without various concentrations of PV (10, 30, and 100 μg/ml) and ACh (30 μM), as a positive control, for 1 h. The supernatants of conditioned cells were deproteinized by zinc-sulfate (30%, v/v) and passed through a copper cadmium reduction column to reduce NO_3_^-^ to NO_2_^-^[25]. As an indicator of NO production, the nitrite concentration in the culture medium was determined using the Griess reagent, as previously described [26]. The culture supernatant (100 μl) was mixed with 100 μl of the Griess reagent (1% sulfanilamide and 0.1% *N*-1-naphthyl ethylenediamine) for 10 min, and then the chromophoric azo-derivative molecule’s absorbance was measured in a microplate reader at 540 nm. Fresh culture medium was used as the blank in all experiments. A range of dilutions of sodium nitrite (NaNO_2_) was used to create a standard curve with the amount of nitrite in each sample. The final NO production was expressed as μmol/l (μM).

### Preparation of total cell lysates

HUVECs (5 × 10^5^ cells/well) in 6-well plates were incubated with or without concentrations of PV (3, 10, and 30 μg/ml) or ACh (30 μM) for 0.5 or 24 h. Total cell lysates were obtained using a lysis buffer (250 mM Tris-HCl (pH 6.8), 1% Triton-100, 0.1% sodium dodecylsulfate (SDS), 1 mM Na_3_VO_4_, 1 mM EDTA, 5 mM sodium fluoride, 1 mM PMSF, and 1 mg/ml leupeptin), and cell debris was removed using a centrifuge at 10,000 × *g* for 10 min at 4°C and stored at -80°C until required. The protein content of the cell lysates was determined using the Bradford assay [27].

### Western blot analysis

Equal amounts of cell lysates (30 μg) were electroblotted onto a nitrocellulose membrane (Millipore), following separation using 8% ~ 12% SDS-polyacrylamide gel electrophoresis (PAGE). The blot was probed using a primary antibody against p-eNOS, total-eNOS (Millipore), Nrf2, HO-1, and β-actin (Santa Cruz Biochemicals, Santa Cruz, CA, USA). The intensity of each band was quantified using density analysis software (Biospectrum 500 Imaging System; Vision Works LS 6.5.2v, UK), and the density ratio represented the relative intensity of each band against controls in each experiment.

### Data and statistical analyses

Results of all experiments are expressed as the mean ± S.E. of multiple experiments (*n* ≥ 3). Data were compared using a one-way analysis of variance (ANOVA) with a post-hoc Bonferroni analysis when applicable, and *p* values of < 0.05 were considered statistically significant. Values of 50% effective concentration (EC_50_) were calculated and obtained from 5 regression lines; each regression line was constructed from 3 ~ 5 points. Values of inhibition of these points ranged 20% ~ 80%.

## Results

### PV-induced rat aortic relaxation

**Figure 1:**
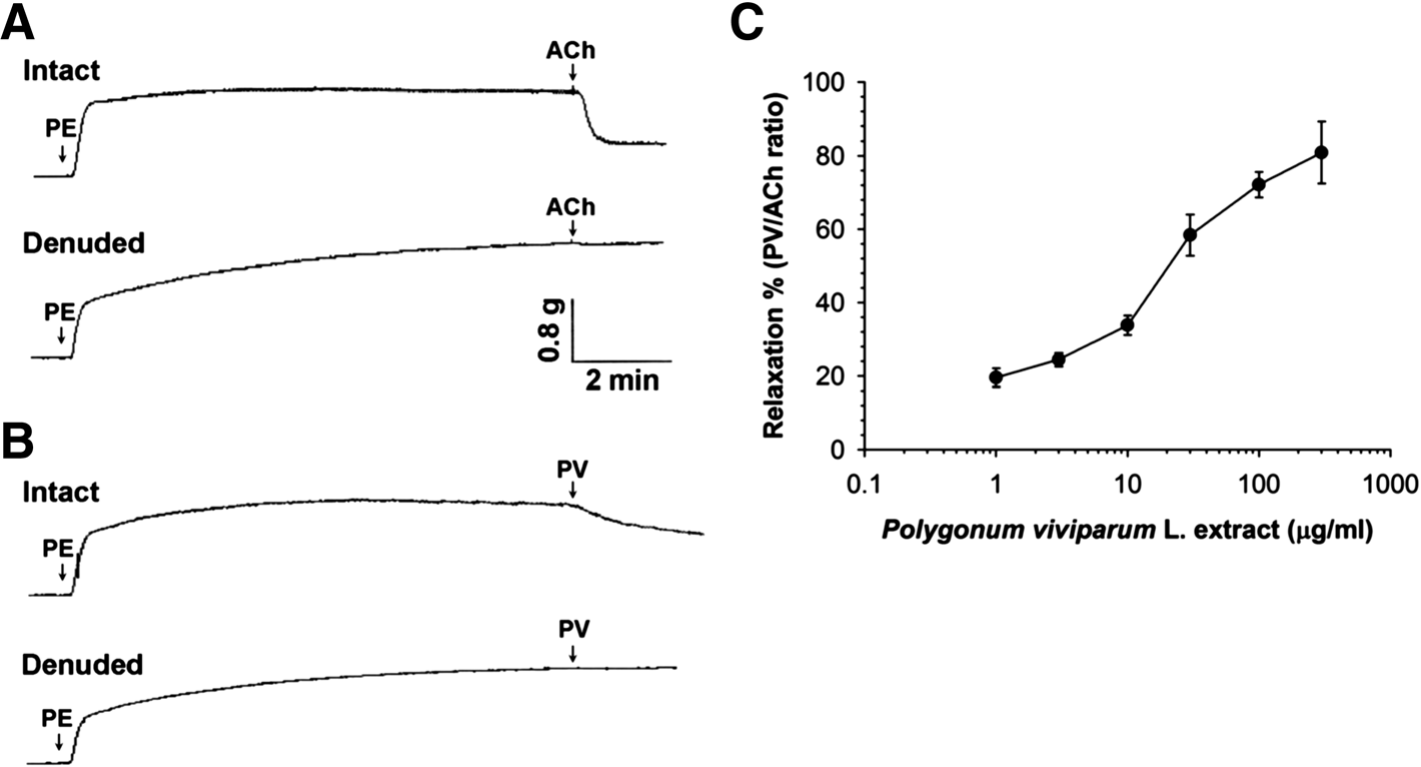
**Relaxation effect of *Polygonum viviparum *L. (PV) in isolated rat thoracic aorta.** The aorta was removed from a rat, and fat and tissue were carefully dissected away in normal 37°C Krebs solution. Phenylephrine (PE) at 3 μM induced a transient phasic contraction followed by tonic contraction, which lasted at least 10 min in intact or endothelial-denuded aorta. Acetylcholine (ACh) at 10 μM induced relaxation in intact (**A**, upper trace) but not denuded (**A**, lower trace) aorta. PV at 100 μg/ml relaxed PE-precontracted intact (**B**, upper trace) but not denuded (**B**, lower trace) aorta. **(C)** Concentration-response curve of vasorelaxation caused by PV on PE-precontracted rat intact aorta. Data are expressed as the mean ± standard error of the mean (S.E.M.) (n ≥ 5 animals per time point).

The vasorelaxing effect of PV was examined on PE (3 μM)-precontracted rat aorta. Data show (Figure 1A) that PE induced a rapid phasic contraction, followed by a tonic contraction lasting for least 10 min in both intact (endothelium-undamaged) and denuded (endothelium-removed) aorta. Treatment with ACh (10 μM) caused relaxation in intact but not denuded aorta. PV relaxed PE-induced contractions in intact but not denuded aorta, as shown in Figure 1B. As shown in Figure 1C, the relaxing responses of PV-induced contractions occurred in a dose-dependent manner. The EC_50_ value was 22.04 ± 1.77 μg/ml. The data indicated that PV might be responsible for the endothelium-dependent vasorelaxation.

### NO synthase (NOS), a guanylyl cyclase inhibitor, and an NO scavenger suppressed PV-induced vasorelaxation

**Figure 2:**
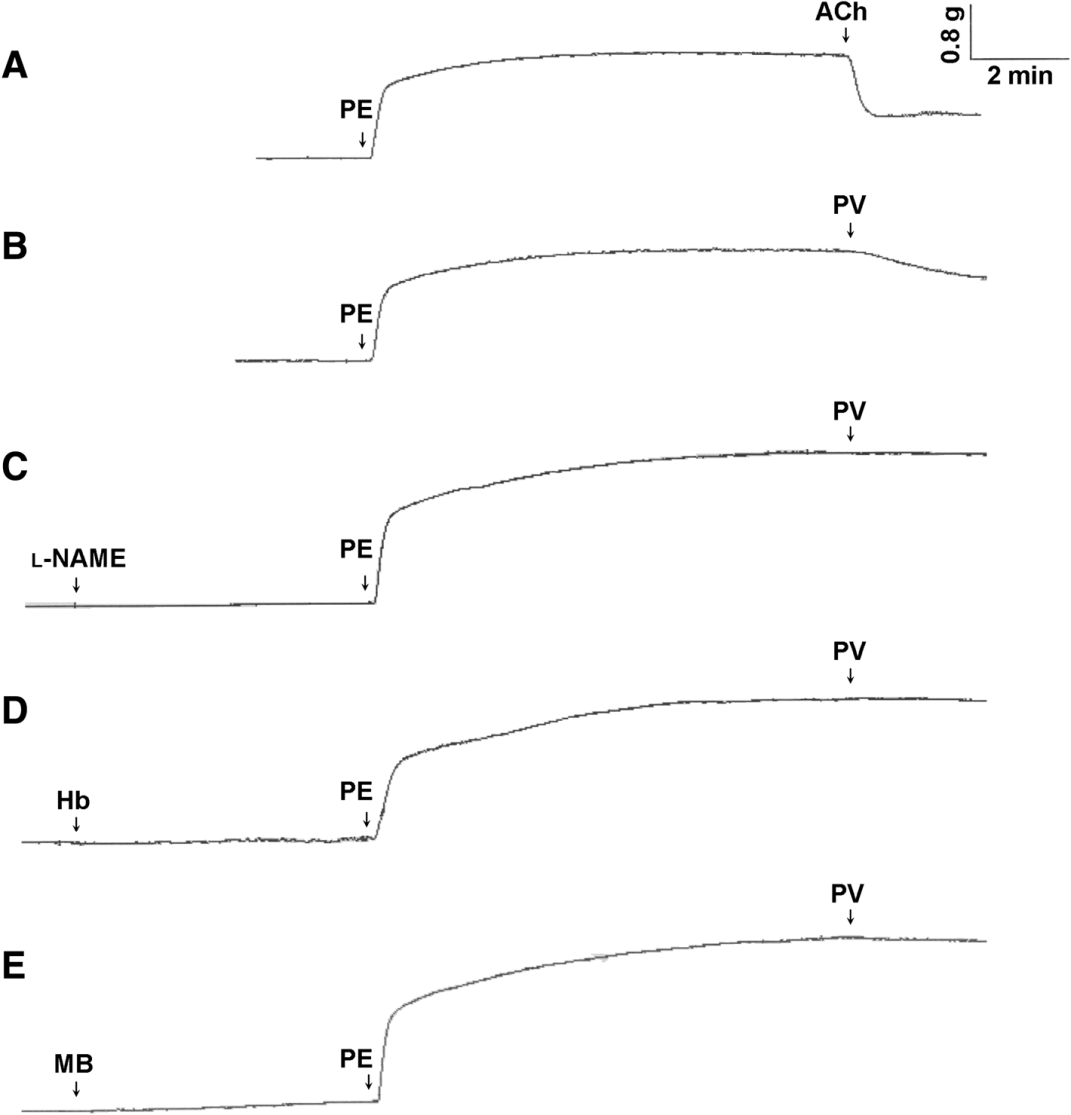
**Effect of an nitric oxide (NO) synthase (NOS) inhibitor, NO scavenger, and guanylyl cyclase inhibitor on *Polygonum viviparum *(PV)-induced relaxation in isolated rat aorta.** Acetylcholine (ACh) at 10 μM and 100 μg/ml PV (traces **A** and **B**, respectively) were treated after 3 μM phenylephrine (PE)-induced vasocontraction reached the maximal force in intact aorta. Prior to the addition of 100 μg/ml PV, the aorta was pretreated with 50 μM l-NAME (trace **C**), 5 μΜ hemoglobin (Hb) (trace **D**), and 10 μM methylene blue (MB) (trace **E**) for 6 min followed by the addition of PE. Each experiments was conducted in aortic rings of at least 5 animals.

To elucidate endothelium-dependent PV-induced vasorelaxation, whether NO is involved in PV-induced vasorelaxation was further investigated. As shown in Figure 2, 10 μM ACh typically relaxed PE-induced vasocontraction in endothelial-intact aorta (trace A). As shown trace B in Figure 2, PV also exhibited vasorelaxation that was similar to ACh’s typical action in intact aorta. Pretreatment with 50 μM l-NAME (an NOS inhibitor) did not influence PE-induced vasocontractions, but PV-induced vasorelaxation was suppressed (trace C). Likewise, as shown in traces D and E, PV-induced vasorelaxation was suppressed by pretreatment with 5 μM Hb (an NO scavenger) and 10 μM MB (a guanylyl cyclase inhibitor).

### Formation of cGMP was elevated by treatment with PV in rat aorta

cGMP formation was measured to confirm whether guanylyl cyclase activation participates in PV-induced vasorelaxation in the rat aorta. Aortic rings were divided into 5 groups: intact, denuded, l-NAME pretreatment, MB pretreatment, and Ca^2+^-free Krebs buffer. As shown in Table 1, the cGMP content of intact aorta significantly increased after treatment with 10 μM ACh and 10 μM SNP. However, ACh did not increase cGMP formation in the denuded aorta. In this experimental model, we found that PV’s effect was similar to that of Ach which induced an increase in cGMP formation in intact but not denuded aorta. In addition, increases in the PV-induced cGMP content were suppressed by pretreatment with 50 μM l-NAME, 10 μM MB, or Ca^2+^-free medium. Therefore, these results further supported that NOS and guanylyl cyclase activities, and extracellular Ca^2+^ are involved in PV-induced vasorelaxation in the aorta.

**Table 1:** **Effects of *Polygonum viviparum *** **(PV) on cyclic guanosine 3′, 5′-monophosphate (cGMP) formation by the rat aorta**

	**cGMP (pmol/mg protein)**				
	**Intact**	**Denuded**	**l-NAME 50 μM**	**MB 10 μM**	**Ca**^**2+ **^**free medium**
Control	0.53 ± 0.08	–	–	–	–
SNP 10 μM	3.45 ± 0.56*	–	–	–	–
ACh 10 μM	3.21 ± 0.42*	0.60 ± 0.18	0.70 ± 0.09	0.52 ± 0.12	0.45 ± 0.11
PV 100 μg/ml	3.18 ± 0.74*	0.57 ± 0.13	0.54 ± 0.09	0.66 ± 0.07	0.64 ± 0.04

After pretreatment with 10 μM IBMX for 5 min, aortic segments were replaced in Ca^2+^-free Krebs’ (2.5 mM EGTA) buffer or treatment with inhibitors of l-NAME and methylene blue (MB) for 10 min and then sodium nitroprusside (SNP), acetylcholine (ACh) and PV were added for another 2 min. The aortic rings were immersed in liquid nitrogen to stop the reaction. cGMP formation were measured. Data are expressed as the mean ± standard error of the mean (S.E.M) of more than 5 individual experiments. –, not determined. * *p* < 0.05 indicates a significant difference from the control.

### PV increased NO production

**Figure 3:**
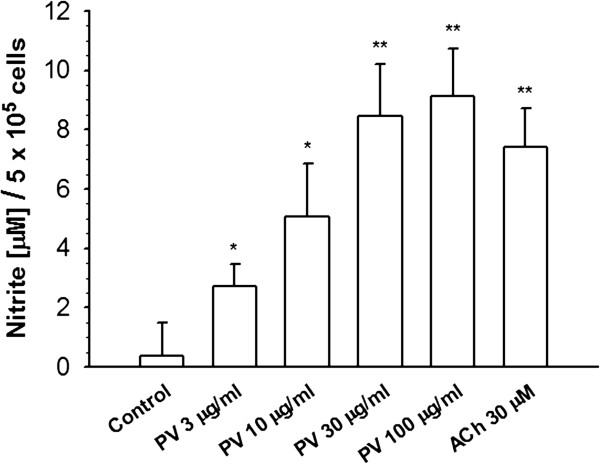
***Polygonum viviparum *(PV) promoted an increase in nitric oxide (NO) production by human umbilical vein endothelial cells (HUVECs).** Cells were treated with 3, 10, 30, and 100 μg/ml PV for 1 h. Medium was then collected for NO detection using the Griess reagent. Data are expressed as the mean ± standard error of the mean (S.E.M.) for more than 5 individual determinations. Treatment with acetylcholine (ACh) was used as a positive control. * *p* < 0.05, ** *p* < 0.01, significantly different from the control.

To confirm NOS’s activity, NO production was directly investigated in cultured medium of HUVECs using the Griess reagent. As shown in Figure 3, NO production dose-dependently increased after treatment with 3, 10, 30, and 100 μg/ml PV in HUVECs for 1 h. ACh was used as a positive control. As shown in Table 2, increases in PV- and ACh-induced NO production were attenuated by treatment with the NOS inhibitors, l-NAME and l-NMMA [28]. In addition, after removal of intercellular Ca^2+^ by treatment with EGTA or chelation of intracellular Ca^2+^ by treatment with 1,2-bis (2-aminophenoxy) ethane-N, N, N’, N’-tetraacetic acid (BAPTA-AM, increases) in PV- and ACh-induced NO production were completely inhibited. These results suggest that influx of extracellular Ca^2+^ is indeed involved in the PV-activated NOS pathway.

**Table 2:** **Effects of *Polygonum viviparum *** **(PV) on nitric oxide (NO) production by human umbilical vein endothelial cells (HUVECs)**

	**Nitrite [μM]/5 × 10**^**5 **^**cells**		
	**Blank**	**PV (30 μg/ml)**	**ACh (30 μM)**
Blank	0.378 ± 0.559	8.445 ± 0.878	7.412 ± 0.661
l-NAME (50 μM)	0.631 ± 0.518	2.073 ± 0.244**	1.066 ± 0.165^##^
l-NMMA (50 μM)	0.476 ± 0.483	2.232 ± 0.383**	1.154 ± 0.397^##^
Ca^2+^-free	0.505 ± 0.442	0.668 ± 0.324**	0.964 ± 0.352^##^
BAPTA-AM (20 μM)	0.432 ± 0.378	0.584 ± 0.460**	0.511 ± 0.369^##^

HUVECs were incubated with PV and acetylcholine (ACh) for 1 h, and then the culture medium was collected for NO detection with the Griess reagent. Ca^2+^-free medium contained 2.5 mM EGTA. BAPTA-AM chelates intracellular Ca^2+^. Data are expressed as the mean ± standard error of the mean (S.E.M) for more than 5 individual experiments. ** *p* < 0.01 indicates a significant difference from PV alone. ^##^*p* < 0.01 indicates a significant difference from ACh alone.

### PV induced eNOS phosphorylation, cytosolic Nrf2 degradation, and HO-1 protein expression

**Figure 4:**
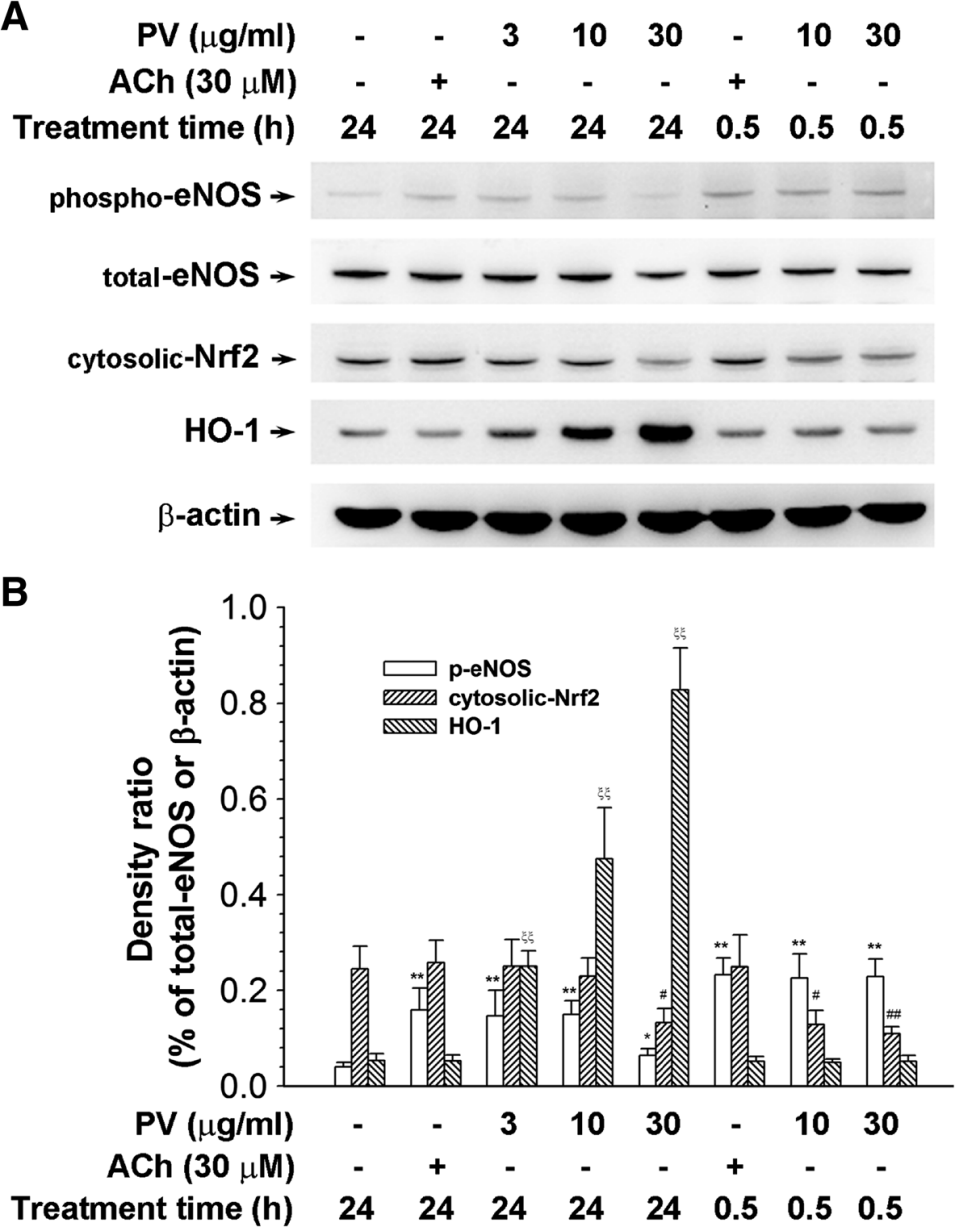
**Effect of *Polygonum viviparum *(PV) on endothelial nitric oxide (NO) synthase (eNOS) phosphorylation, cytosolic nuclear factor E2-related factor 2 (Nrf2) degradation, and heme oxygenase (HO)-1 activation. (A)** Human umbilical vein endothelial cells (HUVECs) were treated with PV (at the indicated concentrations) for 0.5 or 24 h. Total proteins (30 μg) were analyzed on an 8% SDS-PAGE with an antibody specific of eNOS Ser-1177; and analyzed on 10% SDS-PAGE with an antibody specific of Nrf2 and HO-1. **(B)** The relative density was calculated as the ratio of eNOS (phosphor form) expression to eNOS (total form) expression. In addition, the relative density was calculated as the ratio of Nrf2 degradation and HO-1 expression to β-actin expression. Each electrophoretogram is representative of the results from more than 5 individual experiments. * *p* < 0.05, ^#^*p* <0.05, ^ξξ^*p* < 0.01, significantly different from the control.

To elucidate the time coures of PV’s effect in HUVECs, the cells were treated with various concentrations of PV (3, 10, and 30 μg/ml) for 0.5 or 24 h. Cells were harvested by lysis buffer, and total cell lysates were collected; eNOS phosphorylation, cytosolic Nrf2 degradation, and HO-1 protein levels were determined by a Western immunoblot analysis. As shown in Figure 4A, treatment of HUVECs with PV for 30 min resulted in marked phosphorylation of eNOS in a dose-dependent manner, but the total eNOS protein did not increase. However, the phospho-eNOS level slightly decreased after treatment with PV for 24 h compared to treatment with PV for 30 min. Treatment with ACh was used as a positive control. In addition, PV increased cytosolic Nrf2 degradation and HO-1 protein expression. As shown in Figure 4B, it is clear that the density ratio of phosphor-eNOS, cytosolic Nrf2 degradation and HO-1 were elevated by PV in the quantitative determination.

## Discussion

**Figure 5:**
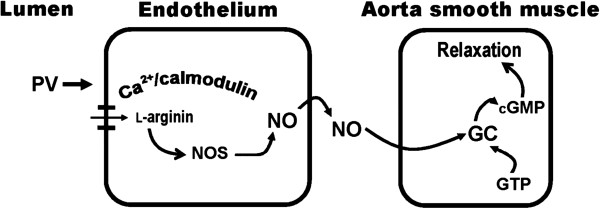
**The flow chart of endothelium-dependent mediator of vasorelaxation by PVin rat thoracic.** PV increase vessel diameter in an endotheliumdependent manner by stimulating production of nitric oxide. cGMP (cyclic guanosine monophosphate); GC (guanylyl cyclase); GTP (guanosine triphosphate); NO (nitric oxide); NOS (nitric oxide synthase).

In this study, effects of PV were investigated on rat aorta ex vivo and on HUVECs in vitro. We explored the potential involvement of eNOS activation in vasorelaxation by PV. First, we found that PV dose-dependently induced vasorelaxation in PE-precontracted aorta in an endothelium-dependent manner (Figure 1). It is well known that NO was first discovered in 1980 as an endothelium-derived relaxing factor [29] and as a key control of vascular homeostasis [30]. NO is a soluble gas and is synthesized from the amino acid l-arginine by a family of enzymes called NOSs [31]. NOS activity can be inhibited by NOS blockers through a mechanism that competes with l-arginine for the substrate-binding site on NOS, such as l-NAME and l-NMMA [32,33], and the NO is caught by NO scavengers, such as Hb [34]. In blood vessels, eNOS converts l-arginine to l-citrulline and NO. Afterwards, NO can diffuse into both the vessel lumen and wall which activates soluble guanylate cyclase, leading to the intracellular accumulation of cGMP [35]. However, soluble guanylate cyclase activity is inhibited by some agents, such as MB [32]. In this study, we found that the vasorelaxation by PV could be suppressed by pretreatment with an NOS inhibitor (l-NAME), a guanylyl cyclase inhibitor (MB), and an NO scavenger (Hb) (Figure 2). These results indicate that the formation of NO and cGMP respectively involves activation of NOS and guanylyl cyclase. Further direct evidence was provided by measuring cGMP in the rat aorta (Table 1). Results showed that PV increased cGMP accumulation, and this effect was suppressed by pretreatment with l-NAME and MB (Figure 5).

Normally, homeostasis of the vessel wall is regulated by endothelial cells that are able to relax vascular smooth muscle cells. Under general physiological conditions, the dominant NOS isoform in the vasculature is eNOS [36]. In order to elucidate whether or not the effect of PV on the rat aorta is through activating eNOS-caused vasorelaxation, we further investigated the effects of PV on HUVECs. We found that NO production significantly increased after treatment with PV in HUVECs, and this increased NO production was inhibited by pretreatment with l-NAME and l-NMMA. In addition, we demonstrated that PV could promote eNOS phosphorylation, cytosolic Nrf2 degradation, and HO-1 protein expression (Figure 4) by HUVECs. It is well known that eNOS can be regulated by multiple phosphorylation sites at tyrosine, serine, and threonine residues [36]. Fulton et al. reported that phosphorylation of Tyr83 regulates the ability of eNOS to produce NO [37]. Heiss et al. reported that activating Nrf2 can elevate the bioavailability of NO by triggering eNOS phosphorylation and reducing eNOS protein expression by HUVECs [38]. The nuclear factor E2-related factor 2 (Nrf2) play a critical role by interacting with cognate DNA-binding domains in the HO-1 promoter to up-regulate *ho-1* gene transcription [39]. Cytoplasmic Nrf2 is bound to the Kelch-like ECHassociated protein 1 under general conditions; however, the Nrf2/Keap1 complex can be disrupted by somecompounds, which allows the translocation of Nrf2 into the nuclei [40]. Previously, we reported that PV can activate the Nrf2/HO-1 pathway to defend against lipopolysaccharide (LPS)-induced macrophage inflammation [4]. Now, we provide evidence that PV can induce Nfr2 activation and further promote eNOS phosphorylation by HUVECs. In the context of the cardiovascular system, knowledge that Nrf2 possesses antioxidant and anti-inflammatory characters can be of benefit in the onset of endothelial dysfunction [41]. Ideally, eNOS is sufficiently phosphorylated to produce NO, and it then has a protective physiological function and reaches its signaling target, mainly activating soluble guanylyl cyclase and eliciting cGMP production in the vasculature [42].

## Conclusions

Results from this study showed that PV can relax PE precontractions in an endothelium-dependent manner in the rat aorta. The fact that activating Ca^2+^/calmodulin-dependent NO synthesis signaling plays a critical role in the regulation of PV-induced vasorelaxation supports our results. Overall, we provide possible mechanistic insights of PV in an approach for therapy of vascular-associated disorders.

## Competing interests

The authors declare that they have no competing interests.

## Authors’ contribution

MLC and JSC contributed equally to this work. MLC, JSC and CMH participated in the design and coordination of the study, carried out the analyses, and wrote the manuscript. WYY, KPC and JSL helped draft the manuscript and analyzed data for the statistical analysis. HWC supplied the sample and related information of Polygonum viviparum L. CMH participated in data interpretation and manuscript preparation. All authors read and approved the final manuscript.

## Pre-publication history

The pre-publication history for this paper can be accessed here:

http://www.biomedcentral.com/1472-6882/14/150/prepub
